# LncRNA SNHG7 sponges miR-216b to promote proliferation and liver metastasis of colorectal cancer through upregulating GALNT1

**DOI:** 10.1038/s41419-018-0759-7

**Published:** 2018-06-18

**Authors:** Yujia Shan, Jia Ma, Yue Pan, Jialei Hu, Bing Liu, Li Jia

**Affiliations:** College of Laboratory Medicine, Dalian Medical University, 116044 Dalian, Liaoning Province China

## Abstract

Accumulating evidence suggests long noncoding RNAs (lncRNAs) play an important role in cancer progression. However, the function of lncRNA SNHG7 in colorectal cancer (CRC) remains unclear. In this study, SNHG7 expression was significantly upregulated in CRC tissues, especially in aggressive cases. In accordance, high level of SNHG7 was observed in CRC cell lines compared to normal colon cells. Furthermore, SNHG7 overexpression promoted the proliferation, migration, and invasion of CRC cell lines, while SNHG7 depletion inhibited invasion and cell viability in vitro. Mechanistically, knockdown of SNHG7 inhibited GALNT1 and EMT markers (E-cadherin and Vimentin). Importantly, SNHG7 directly interacted with miR-216b and downregulation of miR-216b reversed efficiently the suppression of GALNT1 induced by SNHG7 siRNA. Moreover, overexpression of SNHG7 significantly enhanced the tumorigenesis and liver metastasis of SW480 cells in vivo. SNHG7 positively regulated GALNT1 level through sponging miR-216b, and played an oncogenic role in CRC progression. Together, our study elucidated the role of SNHG7 as an miRNA sponge in CRC, and shed new light on lncRNA-directed diagnostics and therapeutics in CRC.

## Introduction

Colorectal cancer (CRC) is the second leading cause of cancer mortality worldwide^1^. This death rate is mainly caused by distant metastasis^2^. Consequently, one well understanding of the molecular mechanism in metastatic CRC is essential for the development of effective treatment strategies in CRC.

NcRNAs are composed of microRNAs (miRNAs) and long noncoding RNAs (lncRNAs). MiRNA targets its seed sequence (5′ end 2–7 nucleotides) to the 3′ untranslated regions (UTRs) of mRNA, leads to mRNA degradation and plays a key role in translation inhibition^3, 4^. LncRNA is a kind of non-encoding RNA transcripts >200 nucleotides in length^5^, many of which show cell type-specific expression^6, 7^. Emerging studies have shown that lncRNAs play important role in cellular development, differentiation, and disease including cancer^8^. RNA transcripts contain miRNA response elements that share miRNAs to communicate with and coregulate each other by titrating the availability of miRNAs, which function as competing endogenous RNAs (ceRNAs) or natural miRNA sponges. LncRNAs participate in the network of ceRNAs in human diseases^9^. SNHG7 (small nucleolar RNA host gene 7) is located on chromosome 9q34.3, with a length of 2157 bp. Recent studies have revealed that SNHG7 expression is overregulated in several cancers such as breast cancer^10^, lung cancer^11^, and malignant pleural mesothelioma^12^. However, the miRNA sponge role of SNHG7 in CRC has not been reported yet.

*N*-acetylgalactosaminyltransferase 1 (GALNT1) is a member of the family of polypeptide *N*-acetylgalactosamine (GalNAc)-transferases (GALNTs). GALNTs initiate mucin-type *O*-linked glycosylation in the Golgi apparatus by catalyzing GalNAc to serine and threonine residues on the target protein^13, 14^. The short core region *O*-glycans are known as cancer-associated Tn-antigens^15^. Altered level of GALNT enzymes have been reported to associate with many cancers. Upregulation of GALNT7 is associated with proliferation, migration, and invasion of cervical cancer cells^16^. GALNT2 downregulation contributes to hepatocellular carcinoma cell malignant behavior^17^. MiR-196b-5p promotes CRC metastasis, at least in part, through the regulation of GALNT5^18^. The expressional change of GALNT1 is associated with many cancers such as bladder cancer, breast cancer, and ovarian cancer^19–21^. However, little is known about the role of GALNT1 in CRC.

Collectively, in this study, we identified that SNHG7 overexpression was a characteristic molecular change in CRC and explored the biological role of SNHG7 on CRC progression. Furthermore, mechanistic analysis revealed that SNHG7 positively regulated GALNT1 expression through sponging miR-216b, thus playing an oncogenic role in CRC pathogenesis. The SNHG7/miR-216b/GALNT1 axis might be a promising therapeutic target for CRC.

## Results

### SNHG7 is upregulated in CRC tissues and cell lines and is predominantly localized in the cell cytoplasm

**Fig. 1:**
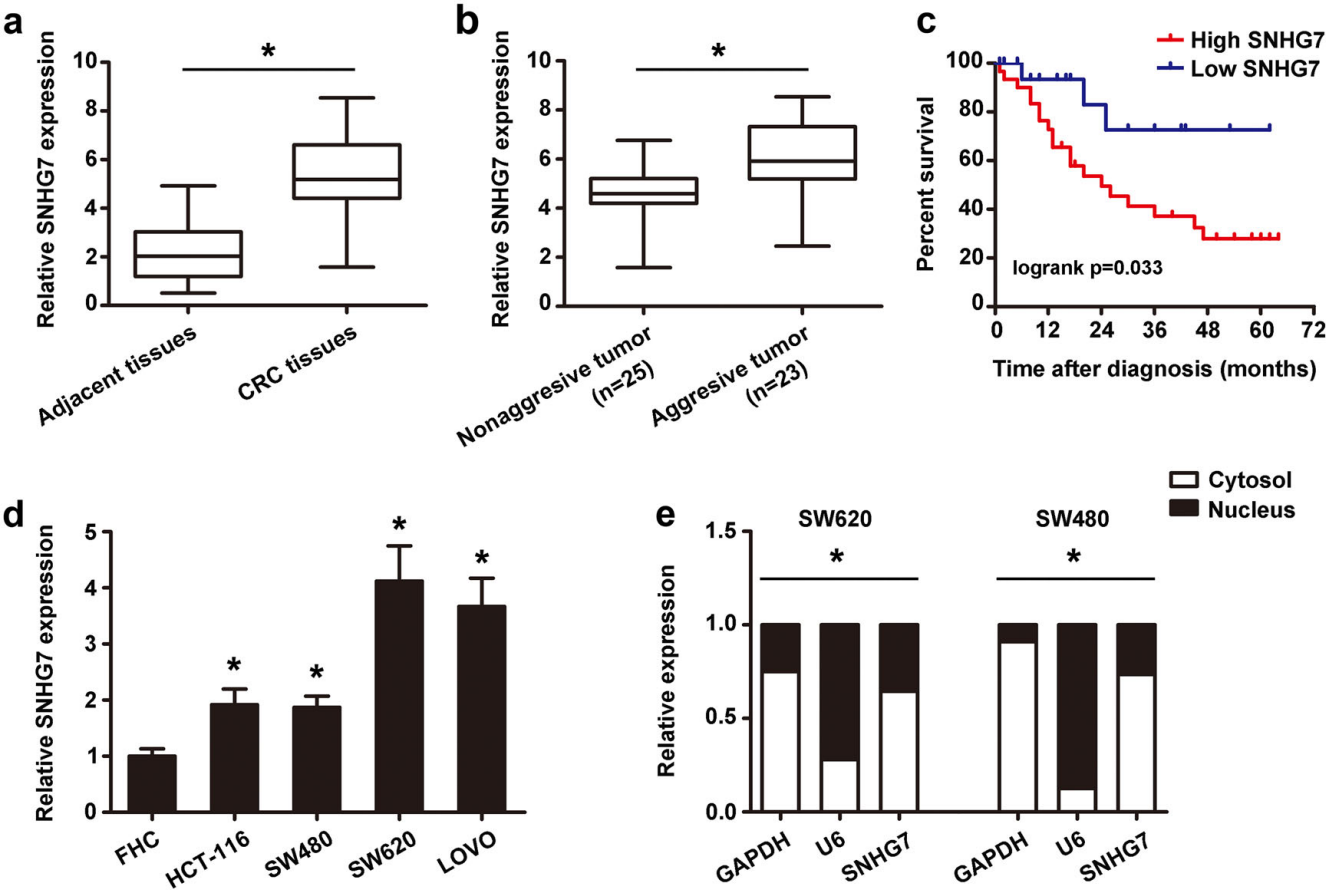
**The differential expression of SNHG7 in CRC tissues and cell lines and subcellular location.** **a** The differential expression of SNHG7 in CRC samples (*n* = 48) and adjacent normal colon tissues (*n* = 48) was shown. **b** The differential expression of SNHG7 in CRC patients with liver metastasis (*n* = 23) and without metastasis (*n* = 25) was analyzed. **c** Kaplan−Meier analyses of the correlations between SNHG7 level and overall survival of 48 patients with CRC (*p* = 0.033; log-rank test). The median of the dataset is selected as the cutoff point between “High SNHG7” and “Low SNHG7”. **d** The differential levels of SNHG7 in CRC cell lines and FHC cell were examined. **e** Cellular localization of SNHG7 in CRC cells was shown. GAPDH and U6 served as a cytoplasmic and nuclear localization marker, respectively. The error bars in all graphs represented SD, and each experiment was repeated three times. *p < 0.05

To investigate the role of lncRNA in CRC metastasis, we analyzed the expressional profiles of lncRNA in SW620 cells (high metastatic cell lines) and SW480 cells (low metastatic cell lines). The microarray analysis showed that SNHG7 level was significantly higher in SW620 cells compared with SW480 cells (data not be shown). To further validate the increase in SNHG7, qRT-PCR analysis was used to investigate the SNHG7 level in 48 pairs of CRC samples and adjacent histologically normal colon tissues (Fig. 1a). Besides, the expression of SNHG7 in 34 fresh CRC samples was evaluated (Fig. S1a and S1b). In the Cancer Genome Atlas (TCGA), SNHG7 exhibited significantly differential abundance when comparing primary tumors with normal colon tissues (Fig. S1c). These data indicated that the expression of SNHG7 in CRC tissues was significantly higher than that in normal tissues. In addition, the SNHG7 expression were obviously higher in CRC patients with liver metastasis than in patients without metastasis (*p* <0.05) (Fig. 1b). Furthermore, correlation analysis revealed that the high level of SNHG7 was significantly associated with clinical stage (*p* = 0.013), lymph node metastasis (*p* = 0.017), and distant metastasis (*p* = 0.009) (Table 1). Kaplan−Meier survival analysis showed that high expression of SNHG7 was associated with CRC poor overall survival (*p* <0.05, Fig. 1c). Interestingly, from TCGA database, we also found that SNHG7 was associated with overall survival of colon adenocarcinoma (COAD) patients (Fig. S1d). High levels of SNHG7 were also detected in SW620, SW480, LOVO and HCT-116 cell lines as compared with FHC cells, suggesting that SNHG7 was upregulated in metastatic cell lines compared with primary cell lines (Fig. 1d). Since cellular location could dictate function of lncRNA, cellular fractionation was performed to identify the subcellular localization of SNHG7 in SW620 and SW480 cells. The results confirmed that SNHG7 was preferentially localized to the cell cytoplasm (Fig. 1e). These data showed that SNHG7 was indeed highly expressed in CRC and was associated with CRC metastasis.

**Table 1:** Correlation between SNHG7 expression and clinicopathological features of colorectal cancer patients

Characteristics	Number of case	SNHG7 expression		
		High	Low	*p* value
	48	30	18	
Age (years)				0.500
<60	27	18	9	
>60	21	12	9	
Gender				0.412
Male	25	17	8	
Female	23	13	10	
Tumor size				0.454
<5cm	26	15	11	
≥5cm	22	15	7	
Tumor stage				0.013*
T1,T2	21	9	12	
T3,T4	27	21	6	
Lymphatic metastasis				0.017*
Absent	24	11	13	
Present	24	19	5	
Distant metastasis				0.009*
Absent	25	20	5	
Present	23	10	13	

Tumor stage was obtained according to the TNM criteria

*p   < 0.05

### Knockdown of SNHG7 inhibits cell proliferation, migration and invasion, and promotes apoptosis of CRC cells in vitro

**Fig. 2:**
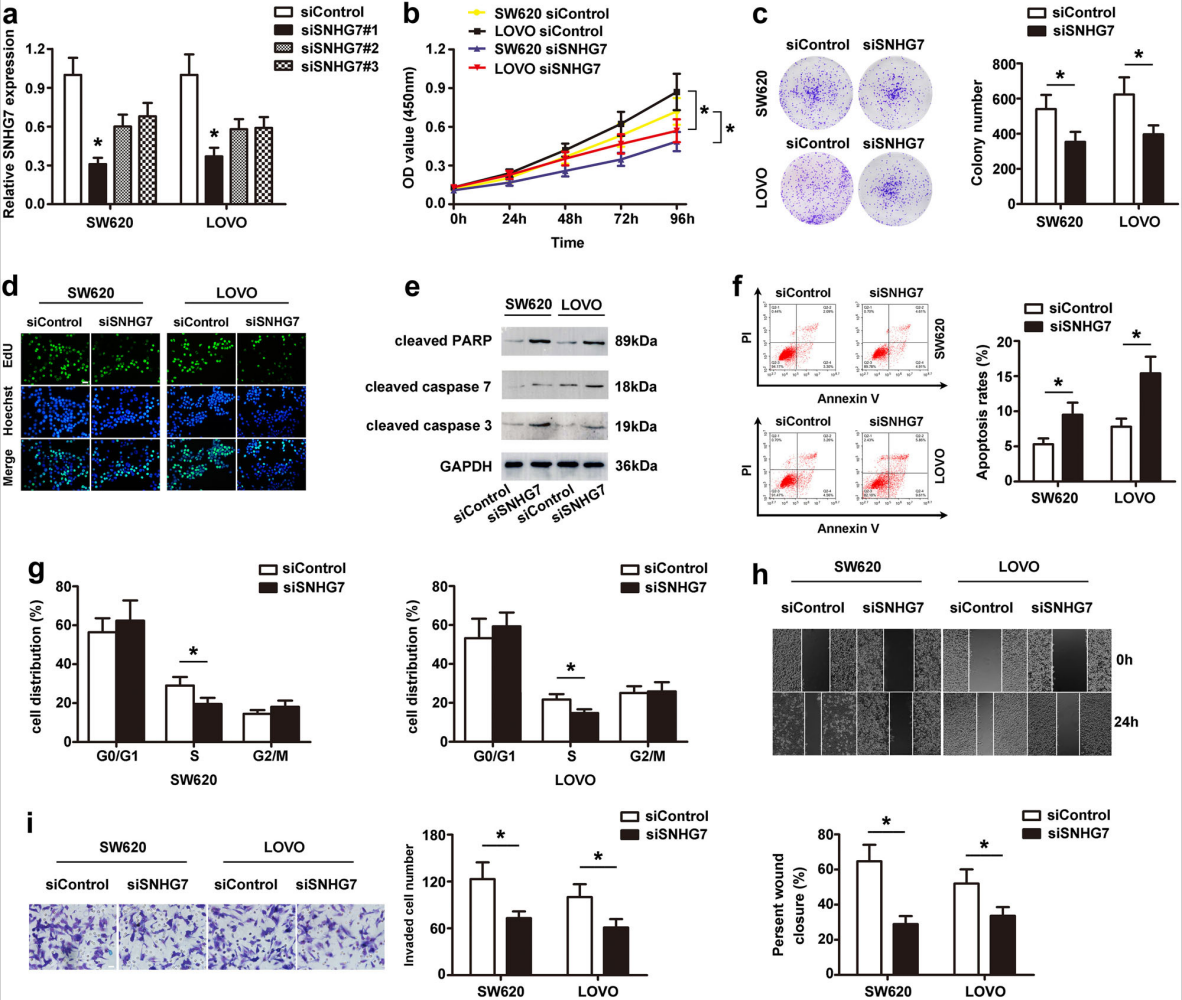
**Knockdown of SNHG7 inhibited cell proliferation, migration and invasion, and promoted apoptosis of CRC cells in vitro.** **a** The level of SNHG7 transfection with siSNHG7 or siControl was analyzed by qRT-PCR. **b** Growth curves of SW620 and LOVO cells after transfection with siSNHG7 or siControl were determined via CCK-8 assays. **c** Colony formation assays showed that knockdown of SNHG7 inhibited CRC cell proliferation. **d** Suppression of SNHG7 expression attenuated the proliferation of CRC cells by EdU assay. Scale bars = 50 μm. **e** The levels of cleaved PARP, cleaved caspase-7, and cleaved caspase-3 following SNHG7 silenced in CRC cells were determined via western blot. **f** Flow cytometry assay showed that silencing of SNHG7 increased the rate of apoptosis in CRC cells. **g** Flow cytometry assay showed that siSNHG7 resulted in S arrest in CRC cells. The cell cycle distribution was exhibited. **h** siSNHG7 resulted in a slower closing of scratch wound by wound-healing assay. Scale bars = 50 μm. **i** Transwell invasion assay was measured and the results were expressed as the number of invaded cells per field. Scale bars = 20 μm. The error bars in all graphs represented SD, and each experiment was repeated three times. *p < 0.05

To examine whether inhibition of SNHG7 could affect the biologic activity of CRC cells, three siRNAs targeting the coding region of SNHG7 (siSNHG7) were tested for their knockdown efficiency. SiSNHG7#1 was the most efficient to be selected for further experiments (Fig. 2a). The CCK-8, colony formation, and EdU assay demonstrated that downregulated expression of SNHG7 attenuated the proliferation of SW620 and LOVO cells (Fig. 2b−d). Furthermore, knockdown of SNHG7 led to increased protein levels of cleaved poly (ADP-ribose) polymerase (PARP), cleaved caspase-7, and cleaved caspase-3 (Fig. 2e). In addition, we observed significantly increased percentage of apoptotic cells (Fig. 2f) in CRC cells-transfected siSNHG7 compared with cells-transfected siControl. Meanwhile, the cell cycle assay demonstrated that the proportion of S markedly declined after knockdown of SNHG7 expression in SW620 and LOVO cells (Fig. 2g). The wound-healing assay showed that cells-transfected siSNHG7-1 underwent a slower closing of scratch wound compared with the siControl group (Fig. 2h). The invasion of SW620 and LOVO cells was significantly reduced following downregulation of SNHG7 expression as shown by transwell assay (Fig. 2i). These data showed that knockdown of SNHG7 could impede CRC cell growth and metastasis.

### Exogenetic overexpression of SNHG7 promotes CRC cells proliferation, migration and invasion, and inhibits its apoptosis in vitro

**Fig. 3:**
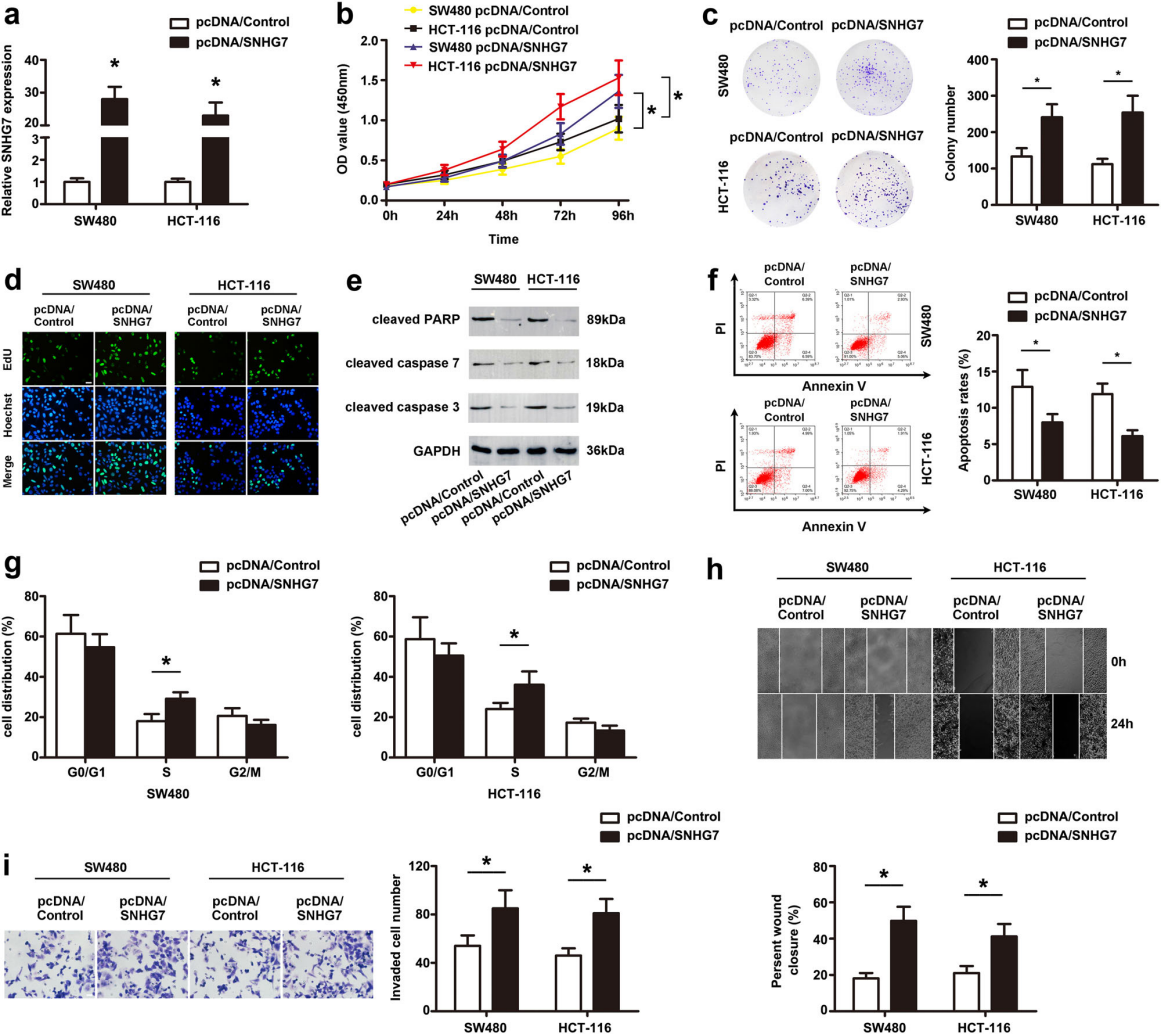
**Overexpression of SNHG7 promoted CRC cells proliferation, migration and invasion, and inhibited apoptosis in vitro.** **a** The expression of SNHG7 in CRC cell lines transfection with pcDNA/SNHG7 or pcDNA/Control was analyzed by qRT-PCR. **b** The proliferative ability of SW480 and HCT-116 cells transfected with pcDNA/SNHG7 or pcDNA/Control was performed by CCK-8 assay. Colony formation (**c**) and EdU assay (**d**) were performed in SW480 and HCT-116 cells transfected with pcDNA/SNHG7 or pcDNA/Control. Scale bars = 50 μm. **e** The expression of cleaved PARP, cleaved caspase-7, and cleaved caspase-3 was analyzed by western blot. **f** The apoptotic rates of cells were detected by flow cytometry. **g** The cell cycle progression of SW480 and HCT-116 cells was evaluated after transfection with pcDNA/SNHG7 or pcDNA/Control using Flow cytometry. **h** Wound scratch assay in pcDNA/SNHG7 or pcDNA/Control transfected SW480 and HCT-116 cells was shown. Scale bars = 50 μm. **i** Transwell invasion assay in pcDNA/SNHG7 or pcDNA/Control transfected SW480 and HCT-116 cells was shown. Scale bars = 20 μm. The error bars in all graphs represented SD, and each experiment was repeated three times. *p < 0.05

To further investigate the role of SNHG7 in progression of CRC cells, SW480 and HCT-116 cell lines with low expression of SNHG7 were used in the following experiments. SNHG7 level was significantly elevated after transfecting with pcDNA/SNHG7 in SW480 and HCT-116 cells (Fig. 3a). Functionally, overexpression of SNHG7 increased the proliferation of SW480, HCT-116 cells by CCK-8 assay (Fig. 3b). Also, colony formation and EdU assays showed that SNHG7 upregulation enhanced the proliferative potential of SW480 and HCT-116 cells (Fig. 3c, d). The levels of cleaved PARP, cleaved caspase-7, and cleaved caspase-3 were markedly decreased in the SNHG7-overexpressed groups compared to control (Fig. 3e). Moreover, the percentage of apoptotic cells was obviously decreased in the overexpressed group of SNHG7 compared with the control (Fig. 3f). The proportion of S was markedly increased after SNHG7 overexpression (Fig. 3g). The migratory and invasive ability was enhanced in cells transfected with SNHG7 by wound-healing and transwell assays (Fig. 3h, i). These data indicated that overexpression of SNHG7 promoted CRC cell growth and metastasis.

### SNHG7 acts as a ceRNA by sponging miR-216b and indirectly regulates GALNT1 expression

**Fig. 4:**
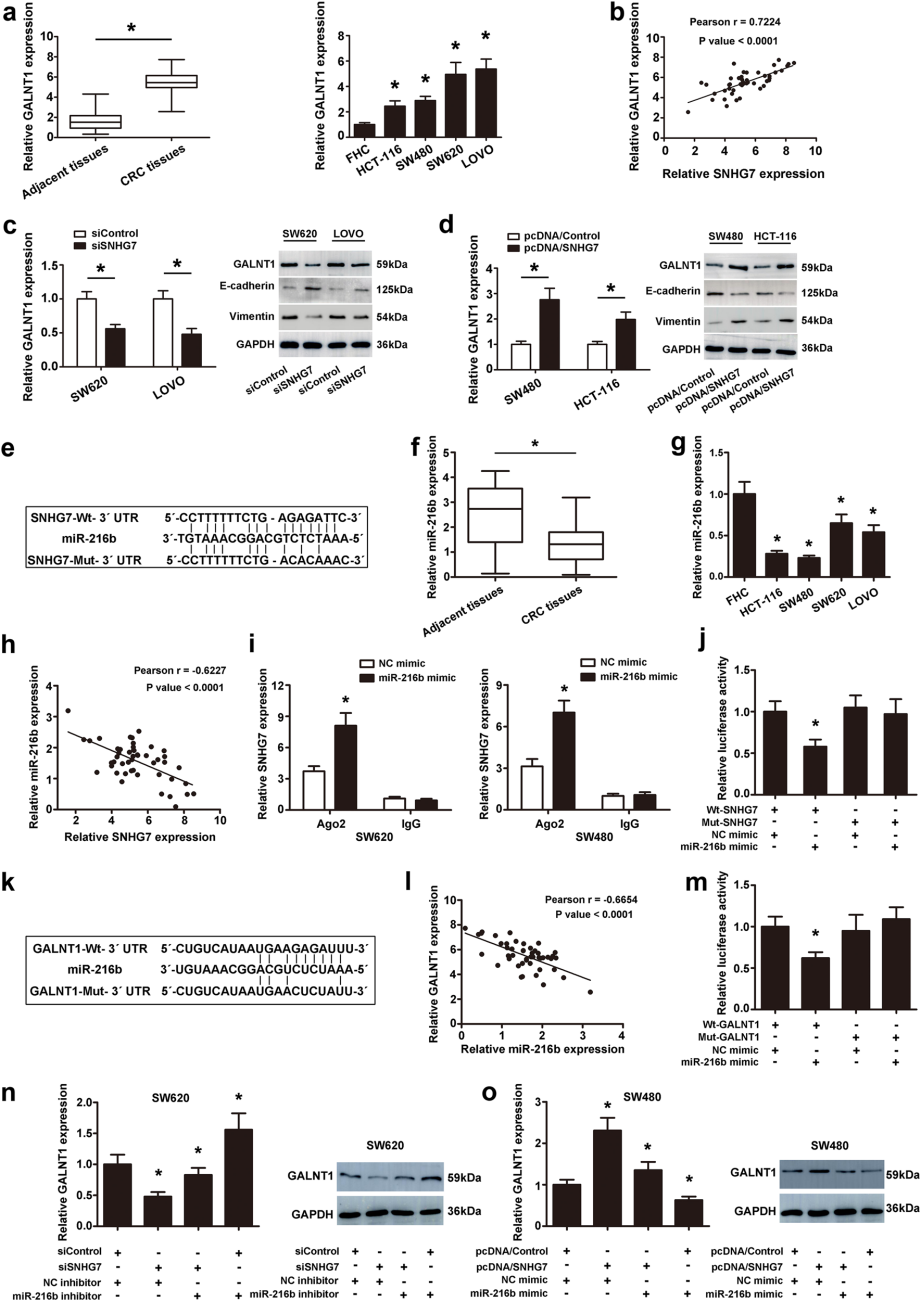
**SNHG7 acted as a ceRNA by sponging miR-216b and regulated GALNT1 expression indirectly.** **a** The differential expression of GALNT1 in CRC tissues and adjacent normal colon tissues was examined. Expressional levels of GALNT1 in CRC cell lines and FHC cell. **b** Pearson’s correlation curve identified positive correlation between SNHG7 and GALNT1 in CRC tissues. **c** The expressional levels of GALNT1 mRNA and protein, E-cadherin and Vimentin proteins in CRC cells transfected with siSNHG7 were evaluated by qRT-PCR and western blot. **d** The levels of GALNT1 mRNA and protein, E-cadherin and Vimentin proteins in CRC cells transfected with pcDNA/SNHG7 were evaluated by qRT-PCR and western blot. **e** The predicted binding sites of miR-216b to the SNHG7 sequence were shown. **f** The differential expression of miR-216b in CRC tissues and adjacent normal colon tissues was analyzed. **g** The levels of miR-216b was investigated in CRC cell lines and human normal colon cell line by qRT-PCR. **h** Pearson’s correlation curve revealed the negative relevance between SNHG7 and miR-216b expression. **i** RNA-IP was performed in SW620 and SW480 cells transfected with NC mimic and miR-216b mimic. SNHG7 expression was detected using qRT-PCR. **j** Luciferase activity of 293T cells cotransfected with miR-216b mimic and luciferase reporters containing SNHG7-Wt or SNHG7-Mut transcript were analyzed. **k** The predicted binding sites of miR-216b to the GALNT1 sequence were shown. **l** Pearson’s correlation curve revealed the negative relevance between GALNT1 and miR-216b levels. **m** Luciferase activity of 293T cells cotransfected with miR-216b mimic and luciferase reporters containing GALLNT1-Wt or GALNT1-Mut transcript were performed. **n** The levels of GALNT1 transfected with miR-216b inhibitor or siSNHG7 in SW620 cell were analyzed by qRT-PCR and western blot. **o** The levels of GALNT1 transfected with miR-216b mimic or pcDNA/SNHG7 in SW480 cells were analyzed by qRT-PCR and western blot. The error bars in all graphs represented SD, and each experiment was repeated three times. *p < 0.05

Gene co-expression network was constructed to visually study the relationship between the validated lncRNAs and mRNAs. According to the network, SNHG7 was highly correlated with GALNT1 (data not shown). GALNT1 expression was much higher in CRC tissues compared in normal adjacent colon tissues. GALNT1 was also highly expressed in the metastasis cell lines than the primary cell lines (Fig. 4a). Furthermore, the SNHG7 and GALNT1 expression showed a significant positive correlation in CRC patients by Spearman’s correlation analysis (Fig. 4b). In SW620 and LOVO cell lines, the mRNA level of GALNT1 was significantly decreased in cells transfected with siSNHG7 compared with control cells (Fig. 4c). Our results revealed that SNHG7 was associated with CRC metastasis, so the markers of EMT (E-cadherin and vimentin) were examined. After inhibiting SNHG7 expression, the protein levels of GALNT1 and Vimentin were decreased, and E-cadherin expression was increased in SW620 and LOVO cell lines (Fig. 4c). On the contrary, the expression of GALNT1 mRNA and protein, and Vimentin protein were upregulated, and E-cadherin expression was downregulated after SNHG7 overexpression in SW480 and HCT-116 cell lines (Fig. 4d).

The previous data showed that SNHG7 was localized preferentially in the cytoplasm. Cytoplasmic lncRNAs could directly bind to miRNA and function as ceRNAs or sponges^22^. Through the bioinformatic database (Starbase v2.0 and miRcode), we predicted the potential miRNA binding sites in SNHG7, which was among the numerous possible targets of miR-216b (Fig. 4e). MiR-216b level was much lower in CRC tissues than in normal adjacent tissues by qRT-PCR. MiR-216b was also lowly expressed in the CRC cell lines than the normal colon cell lines (Fig. 4f, g). Spearman’s correlation analysis suggested a significant negative correlation between SNHG7 and miR-216b in CRC tissues (*r* = −0.6039, *p* <0.0001, Fig. 4h). In order to further verify the direct binding between miR-216b and SNHG7 at endogenous levels, anti-Ago2 RIP assay was performed in SW620 and SW480 cells transiently overexpressing miR-216b. Compared with the NC group, the endogenous SNHG7 was specifically enriched in the cells of miR-216b mimic transfected group (Fig. 4i), indicating that SNHG7 directly targeted miR-216b. Then a dual-luciferase reporter assay was performed to further verify the surmise that SNHG7 was directly targeted by miR-216b in 293T cells. The luciferase activity was discovered to be pronouncedly reduced when wild-type SNHG7 3′-UTR and miR-216b mimic were cotransfected into 293T cells in comparison with the activity in 293T cells cotransfected with wild-type SNHG7 3′-UTR and miR-216b control. However, the luciferase activity of Mut-SNHG7 showed no statistical changes (Fig. 4j).

GALNT1 was initially identified as potential miR-216b targeted gene using public prediction algorithms (miroRNA.org and Starbase v2.0) (Fig. 4k). As shown in Fig. 4l, Spearman’s correlation analysis assumed that GALNT1 and miR-216b had a negative correlation. Dual-luciferase reporter assay showed that GALNT1 was also the direct target of miR-216b (Fig. 4m). As shown in Fig. 4n, o, GALNT1 expression was significantly decreased after transfecting with siSNHG7 and NC inhibitor. The inhibitory effect of siSNHG7 was notably reversed by co-transfection with siSNHG7 and miR-216b inhibitor in SW620 cells. Otherwise, the expression of GALNT1 was significantly increased after transfecting with pcDNA/SNHG7 and NC mimic. SNHG7 advance was also reversed by transfection with miR-216b mimic in SW480 cells. The above results suggested that SNHG7 functioned as a ceRNA by sponging miR-216b and indirectly regulated GALNT1 expression.

### MiR-216b reverses the promoting effect of SNHG7 on the growth and metastasis of CRC cells

**Fig. 5:**
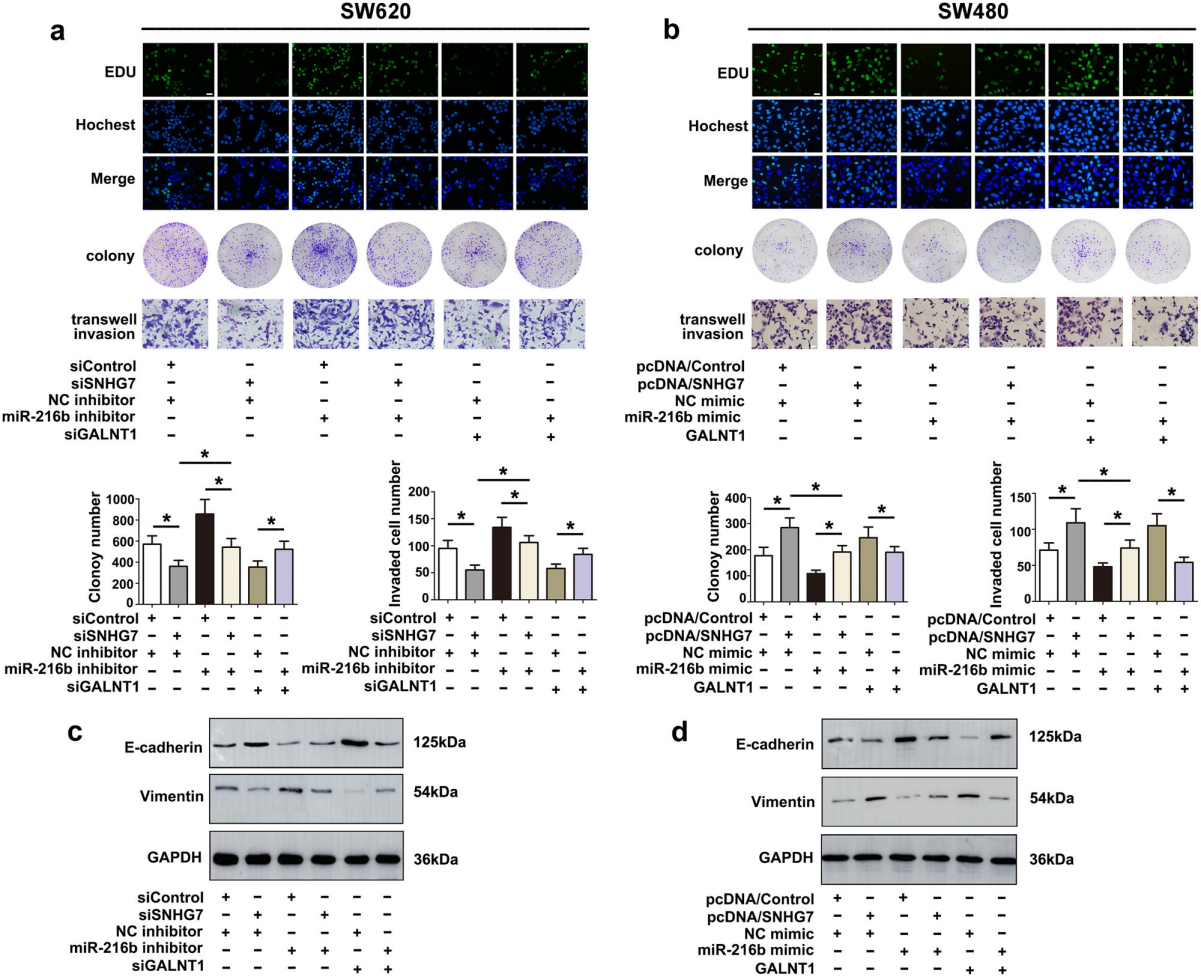
**MiR-216b reversed the promoting effect of SNHG7 on the growth and metastasis of CRC cells.** **a** and **b** Functional assays identified the phenomenon of SNHG7 and GALNT1 regulated each other to compete for the binding of miR-216b by EdU assay, colony formation, transwell invasion assay in SW620 and SW480 cell lines. Scale bars = 50 μm (EDU) and 20 μm (transwell invasion). **c** The levels of E-cadherin, Vimentin in SW620 cells cotransfected siSNHG7 with miR-216b inhibitor and siGALNT1 was analyzed by western blot. **d** The levels of E-cadherin, Vimentin in SW480 cells cotransfected pcDNA/SNHG7 with miR-216b mimic and GALNT1 were examined by western blot. The error bars in all graphs represented SD, and each experiment was repeated three times. *p < 0.05

To determine whether SNHG7 exerted its function through miR-216b in CRC cells, rescue experiments were conducted. Knockdown of the endogenous highly expressed SNHG7 or GALNT1 significantly weakened the proliferation, migration, and invasion of SW620 cells. Suppression of miR-216b blocked the effect induced by SNHG7 or GALNT1 depletion. Moreover, knocking down miR-216b alone dramatically enhanced the proliferative and invasive ability of SW620 cells (Fig. 5a). In contrast, the increased SNHG7 or GALNT1 boosted the proliferative and invasive capacity of SW480 cells. The influence of pcDNA/SNHG7 or pcDNA/GALNT1 was reversed by upregulation of miR-216b in SW480 cells. Furthermore, promoting the expression of miR-216b alone significantly inhibited the proliferative and invasive ability of SW480 cells (Fig. 5b). The expression of EMT-induced markers E-cadherin and Vimentin in transfected CRC cells were analyzed. The influence of siSNHG7 or pcDNA/SNHG7 on the expression was reversed by miR-216b inhibitor or mimic in CRC cells (Fig. 5c, d). These data suggested that miR-216b reversed the promoting effect of SNHG7 on the growth and metastasis of CRC cells.

### Ectopic expression of lncRNA SNHG7 promotes CRC growth and liver metastasis in vivo

**Fig. 6:**
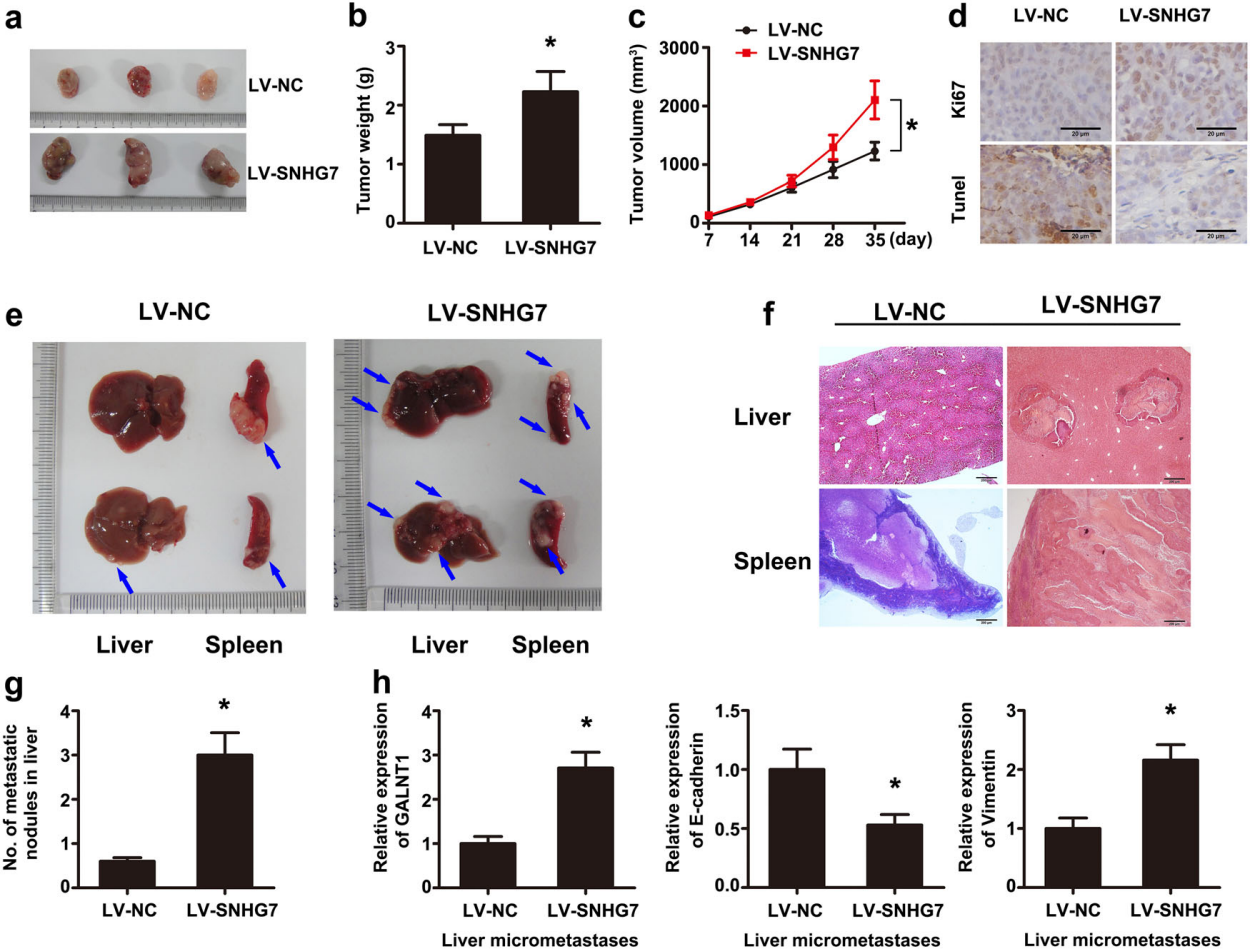
**Ectopic expression of SNHG7 promoted CRC growth and liver metastasis in vivo.** **a** Tumors collected from mice were exhibited. **b** Tumor weight of mouse was measured. **c** Tumor volume curve of mouse upon LV-SNHG7 or LV-NC treatment was analyzed. **d** Immunohistochemical staining of Ki-67 was used to assess proliferation, and apoptosis was detected using Tunnel kit. **e** Representative sections of liver tumors (*n* = 6 in each group) were shown. The blue arrows indicated the tumor nodules. Overexpression of SNHG7 significantly increased the metastasis of SW480 cells to the liver. **f** The liver and spleen sections were shown via HE staining (scale bar = 200 μm). **g** The number of metastasis in the liver was determined. **h** As assessed by qRT-PCR, the levels of GALNT1 and Vimentin mRNA were increased and the level of E-cadherin was decreased, respectively, in liver metastasis originating from mice in the SW480-LV-SNHG7 group compared with that in the SW480-LV-NC group. The error bars in all graphs represented SD, and each experiment was repeated three times. *p < 0.05

In order to research whether SNHG7 caused CRC growth in vivo, we conducted a subcutaneous tumor formation experiment in nude mice. SW480 cells stably overexpressing SNHG7 or empty vector were injected subcutaneously into the nude mice right flank. As shown in Fig. 6a, LV-SNHG7 injected mice showed an addition in tumor weight and volume at the end of the in vivo assay compared to the control group (Fig. 6b, c). Moreover, SNHG7 overexpression promoted tumor proliferation and inhibited cell apoptosis (Fig. 6d).

Next, spleen injection experiment was adopted to determine the effect of SNHG7 on the metastasis of CRC cells in vivo. In contrast with the SW480 LV-NC group, most mice in SW480 LV-SNHG7 group formed more liver metastases (*p* <0.05; Fig. 6e−g). Furthermore, we assessed the levels of GALNT1, E-cadherin, and vimentin in liver metastasis of mice. Compared with the LV-NC group, the mRNA levels of GALNT1 and vimentin were increased in LV-SNHG7 group. E-cadherin expression was decreased in the LV-SNHG7 group compared with the LV-NC group (Fig. 6h).

Moreover, to evaluate the biological function of SNHG7 in CRC lung metastasis, green fluorescently labeled SW480 cells with SNHG7 overexpression compared to negative control were intravenously injected into nude mice via the tain vein. At the beginning to show symptoms of dying after injection, quantified tumor metastasis to lung was performed by in vivo imaging system (Fig. S2). We observed that overexpression of SNHG7 enhanced CRC lung metastasis. Besides, the levels of GALNT1, E-cadherin, and vimentin in mice lung metastasis were evaluated. Interestingly, these results were consistent with our findings in liver metastasis. Collectively, SNHG7 promoted CRC cell growth and metastasis in vivo.

## Discussion

Emerging studies have revealed that lncRNA plays an important role in cancer progression, and abnormal expression of lncRNA has been found in the CRC^23, 24^. For instance, lncRNA HOTAIR was a negative prognostic factor in both primary tumors and the CRC blood^25^. Zhang et al. reported that lncRNA CASC11 could activate the WNT/β-catenin pathway to promote cell growth and metastasis in CRC^26^. Accordingly, in several cancers, lncRNA SNHG7 has been known as a potent oncogene^11^. In this study, we found that SNHG7 level was significantly increased in the CRC tissues and CRC cell lines. In addition, the level of SNHG7 in CRC patients was related to the aggressiveness and survival rate. Functional investigation revealed the tumorigenic role of SNHG7 in promoting cellular proliferation, inhibiting apoptosis, and enhancing metastasis in CRC. In vivo assay, overexpression of SNHG7 could promote CRC cell growth and liver matastasis. Interestingly, SNHG7 was upregulated not only in CRC but also in breast cancer, lung cancer, and malignant pleural mesothelioma. Thus, we proposed that SNHG7 might be an oncogene in CRC.

Glycosylation is frequently altered in cancers and suspected to contribute to the progression of tumors^27^. Some of the most common and striking characteristics of malignant tumors were perturbations to *O*-glycosylation and the altered level of *O*-glycans^28^. There was a significant increase in polypeptide alpha-linked terminal GalNAc of 90% solid breast tumors^29^. GALNT3 and GALNT6 levels were increased in pancreatic cancers^30, 31^. Aberrant glycosylation from GALNT1 mutation was involved in hepatocellular carcinoma, melanoma, and bladder cancers^19, 32, 33^. GALNT1 strongly promoted liver tumor growth after relocating to the endoplasmic reticulum^34^. Whether GALNT1 might act as marker of CRC liver metastasis or potential target for vaccine remained unclear. Here, we showed that GALNT1 expression was increased in metastatic CRC cell lines and tumor tissues. And there was a positive correlation between SNHG7 and GALNT1. Therefore, these findings suggested that GALNT1 might be correlated with CRC progression.

Previously, miR-216b was reported to be abnormally expressed in many cancers, including CRC, hepatocellular carcinoma, human melanoma, and ovarian cancer^35–39^. Moreover, miR-216b widely participated in cell proliferation and invasion, as well as autophagy and apoptosis^40, 41^. HIF-2a-MALAT1-miR-216b axis regulated multidrug resistance of hepatocellular carcinoma cells via modulating autophagy^42^, but little was known about its function in CRC. miRNAs were thought to regulate the expression of a variety of coding genes simultaneously. Thus, we speculated that miR-216b might have different effect on cell phenotype in different cell lines by targeting different target genes. In the present study, we observed an inverse correlation between SNHG7 and miR-216b. SNHG7 bound directly to miR-216b. Importantly, we showed the endogenous interaction between SNHG7 and miR-216b by coimmunoprecipitation with the Ago2 protein in CRC cells. SNHG7 could play the role of endogenous sponge or ceRNA, to control miR-216b availability for its target gene GALNT1. Several other targets of miR-216b, such as MALAT1^42^, FGFR1^43^, Beclin-1^36^, had been reported to involve in cell invasion and migration. In this study, we concentrated on GALNT1, which was highly associated with SNHG7. Ectopic SNHG7 at least partially rescued suppression of the effect of miR-216b on cell proliferation, migration, and invasion. Taken together, these data suggested that SNHG7 might play an oncogenic role via SNHG7/miR-34a/GALNT7 axis.

In summary, overexpressed SNHG7 promoted CRC cell proliferation and metastasis in vitro and in vivo, suggesting that SNHG7 exhibited oncogenic properties in CRC progression. SNHG7 exerted the oncogenic effects partially through SNHG7/miR-216b/GALNT1 axis. These findings provided useful information to find new biomarkers for early diagnosis and therapeutic application in CRC progression.

## Materials and methods

### Patients and tissues

Two independent cohorts were enrolled. Cohort 1: Forty-eight CRC tissues and adjacent tissues were collected from patients, who underwent radical resection at the First Affiliated Hospital of Dalian Medical University during 2012−2016. Tumor and adjacent tissues were snap-frozen in liquid nitrogen immediately after extraction and stored at −80 °C until total RNA was extracted. Cohort 2: Fresh CRC and adjacent normal tissues were collected from 34 patients between March 2015 and December 2017. All the patients were diagnosed by histology and not treated with neoadjuvant therapy. The study was approved by the Ethics Committee of the First Affiliated Hospital of Dalian Medical University (approval number YJ-KY-FB-2016-16), and written informed consent was obtained from all patients.

### Cell culture and transfection

Human CRC cell lines (SW480, SW620, LOVO, and HCT-116) and human normal colon epithelial cell line FHC were purchased from KeyGEN Company (Nanjing, China). SW480 and SW620 cells were cultured in Leibovitz’s L-15 (Gibco, Grand Island, NY) medium supplemented with 10% fetal bovine serum (FBS; Gibco, Grand Island, NY), 1% penicillin–streptomycin (HyClone, Logan, Utah, USA), FHC, HCT-116 and LoVo cells were maintained in 90% RPMI DMEM (Gibco,Grand Island, NY) containing 10% FBS, 1% penicillin–streptomycin at 37 °C in air containing 5% CO_2_.

Scrambled siRNA of SNHG7 or GALNT1 (siControl) and SNHG7 siRNAs (siSNHG7#1, siSNHG7#2 and siSNHG7#3), GALNT1 siRNA (siGALNT1), as well as pcDNA3.1-Control (pcDNA/Control), pcDNA3.1-SNHG7 (pcDNA/SNHG7) and pcDNA3.1-GALNT1 (pcDNA/GALNT1) were purchased from GenePharma (Shanghai, China). MiR-216b inhibitor, inhibitor negative control (NC inhibitor), miR-216b mimic and mimic negative control (NC mimic) were also purchased from GenePharma (Shanghai, China). Lipofectamine 2000 Reagents (Invitrogen Co., Carlsbad, CA, USA) were used for cell transfection. In vivo experiments, full-length SNHG7 cDNA subcloned into LV5 lentiviruses (GenePharma, Shanghai, China) and infection into SW480 cells to generate SW480/LV-SNHG7.

### Dual-luciferase reporter assay

5×10^4^ cells were seeded in 24-well plate overnight. Then, cells were cotransfected with pcDNA3.1 SNHG7-wt or pcDNA3.1 SNHG7-mut into HEK-293T cells together with miR-216b mimic or control. After 24 h, cells were harvested for luciferase detection using the dual-luciferase reporter assay system (Promega, Madison, WI, USA). Experiments were performed three times.

### RNA immunoprecipitation (RIP) assay

The Magna RIP^TM^ RNA Binding Protein Immunoprecipitation Kit (Millipore, USA) was used for RNA immunoprecipitation (RIP) experiments. The RIP assay was used to explore the binding relationship between endogenous SNHG7 and miR-216b in CRC cells, according to the instructions. SW620 and SW480 cells were collected and lysed in RIP lysis buffer, 100 μl of cell lysate was incubated with RIP buffer containing magnetic beads conjugated with a human anti-Argonaute2 (Ago2) antibody (Millipore, USA) or negative control IgG (Millipore, USA). The samples were incubated with proteinase K with shaking to digest proteins and then the precipitation of RNA was obtained. Purified RNA was analyzed to RT-PCR for further study.

### RNA isolation and quantitative real-time PCR analysis

Total RNA from clinical tissue and CRC cells was extracted using TRIzol reagent (Invitrogen, Carlsbad, CA, USA) according to the manufacturer´s instructions. 1 µg of total RNA was reverse transcribed to cDNA using a Reverse Transcription Kit (QIAGEN, Valencia, CA, USA). Real-time PCR analyses were performed using SYBR-Green-quantitative real-time PCR Master Mix kit (Toyobo Co., Osaka, Japan). The mirVanaTM qRT-PCR microRNA Detection Kit (Ambion Inc., Austin, TX, USA) was used for miRNA detection according to the manufacturer’s instructions. A specific stem-loop RT primer was used for miR-216b. The primers sequences used were as follows: SNHG7 forward: 5′-GTGACTTCGCCTGTGATGGA-3′, reverse: 5′-GGC CTCTATCTGTACCTTTATTCC-3′; GALNT7 forward: 5′-GTTGGTGGCCCTGGA GAGA-3′, reverse: 5′-ACAACGCTCGAAGTGAGCAA−3′; Vimentin forward: 5′-CATTGAGATTGCCACCTAC-3′, reverse: 5′-CGTTGATAACCTGTCCATC-3′; E-cadherin forward: 5′-AGAACGCATTGCCACATACA-3′, reverse: 5′-GAGGAT GGTGTAAGCGATGG-3′; U6 forward: 5′-CTCGCTTCGGCAGCACA-3′, reverse: 5′-AACGCTTCACGAATTTGCGT-3′; GAPDH forward: 5′-AACGTGTCAGTGGT GGACCTG-3′, reverse: 5′-AGTGGGTGTCGCTGTTGAAGT-3′. MiR-216b was normalized to U6. SNHG7 and mRNA expression data were normalized to GAPDH. The 2^−△△Ct^ method was used to calculate the fold changes. Each experiment was repeated three times.

### Subcellular fractionation

To determine the cellular localization of SNHG7, nuclear fraction was isolated from cytoplasm according to the manufacturer’s instruction for the PARIS Kit (Life Technologies, Thermo Fisher Scientific, USA). RNA was extracted from the cytoplasm and nucleus of cells respectively. The expression ratio of specific RNA molecules between the cytoplasmic and nuclear fraction was detected by QRT-PCR. GAPDH and U6 act as the cytoplasmic and nucleus control, respectively.

### Western blot

Equal amount protein was separated by SDS-PAGE and transferred to polyvinylidene difluoride membrane (Millipore, Bedford, MA, USA). The membrane was blocked with 5% nonfat dry milk in PBS containing 0.1% Tween 20 (PBST) for 1 h. Then the above PVDF membranes were incubated with corresponding primary antibodies, E-cadherin, (1:2000, Proteintech, Chicago, USA), Vimentin (1:1000, Proteintech, Chicago, USA), GALNT1 (1:250, Abcam, Cambridge, UK), and GAPDH (1:1000, Santa Cruz Biotech, Santa Cruz, CA). The membranes were incubated with HRP-conjugated secondary antibodies (1:1000, Santa Cruz Biotech, Santa Cruz, CA) for 2 h at room temperature. Detection was performed by ECL western blotting kit (Amersham Biosciences, Buckinghamshire, UK). GAPDH was used as the loading control.

### CCK-8 and colony formation assays

CCK-8 assay was used for CRC cell proliferation analysis. CRC cells were seeded in 96-well plate at a density of 1×10^3^ cells per well. After incubation, 10 μl CCK8 were added to the wells at different time. The absorbance was measured at 450 nm by microplate reader (Bio-Rad, Hercules, CA, USA).

Colony formation assay was performed to measure the capacity of cell proliferation. 1.5×10^3^ cells/well of SW480, SW620, HCT-116 and LOVO were seeded in a six-well plate and cultured for 10 days. Colonies were then fixed with 10% formaldehyde for 30 min and stained for 5 min with 0.5% crystal violet. Then colonies were photographed with NIKON camera and analyzed by software IPP Image-Pro Plus 6.0.

### 5-Ethynyl-20-deoxyuridine (Edu) assay

Cell proliferation was also determined by 5-Ethynyl-2’-deoxyuridine assay using a KeyFluor488 Click-iT EdU kit (KeyGEN BioTECH, Nanjing, China). The EdU assay was performed according to the manufacturer’s instruction. The cells were then visualized under a fluorescence microscopy (20 × 10). To assess cell proliferation activity, the ratio of EdU-stained cells (with green fluorescence) to Hoechst-stained cells (with blue fluorescence) was calculated. Experiments were repeated at least three times.

### Flow cytometric analysis

Apoptosis assay was performed using an Annexin-V-FITC apoptosis detection kit (BD, Franklin Lakes, NJ, USA). Briefly, cells were harvested using trypsin, washed twice with ice-cold PBS and resuspended in 100 μl flow cytometry binding buffer. And stained with 5 μl Annexin V/FITC followed by 5 μl PI at the room temperature in the dark for 15 min. Four hundred microliters binding buffer was added to each tube, and the apoptosis cells were analyzed by FACSCalibur flow cytometer (BD Biosciences, CA, USA).

For cell cycle analysis, 1×10^6^ cells were harvested by trypsinization and fixed overnight at −20 °C with ice-cold 70% ethanol. After washing with PBS, the cells were resuspended in propidium iodide (50 μg/ml, PI, Sigma) and RNAseA (0.1 mg/ml, Sigma) and stained at room temperature for 30 min. Then was analyzed by flow cytometer (BD Biosciences, CA, USA).

### Wound-healing assay

4×10^5^ cells were cultured into 12-well plates and grew until 80–90% confluence. The cell monolayer was scratched with a sterile pipette tip. At different times (0, 24 h), the images of cell morphology were captured under an Olympus microscope (10 × 10). The results were analyzed by software IPP Image-Pro Plus 6.0.

### In vitro migration and invasion assays

The migratory and invasive ability of the cells were measured using transwell chambers (Corning, New York, USA). Cells (5×10^4^) suspended in serum-free L-15 were transferred to the upper chamber. The L-15 medium containing 10% FBS was added as chemokine in the lower chamber. After 24 h, the migrated cells on the membrane lower surface were fixed with 75% methanol, and stained with crystal violet. Evaluation of invasive capacity was performed by counting invading cells under a microscope (40 × 10), and five random fields of view were analyzed for each chamber. All experiments were performed in triplicate.

### In vivo proliferation and metastasis assays

Male BALB/c nude mice (4–5 weeks old) were purchased from the Model Animal Research Center of Nanjing University and maintained in a specific pathogen-free facility. The protocol was approved by the responsible governmental animal ethics committee.

To evaluate the in vivo tumorigenic effects, SW480-Lv-SNHG7 and SW480-Lv-NC cells (2×10^6^) were inoculated subcutaneously in the right flank of the nude mice. Tumors were measured every 7 days and tumor volume was calculated. Five weeks after CRC cells inoculation, the mice were killed and the tumors were collected.

For the hepatic metastasis model, mice were anaesthetized with pentobarbital sodium (Sigma, USA) by intraperitoneal injection. A 1-cm incision was formed on the left side and the spleen was separated, a total of 2×10^6^ CRC cells were suspended in PBS and then injected into the spleen with a 30-gauge needle. The spleen was returned to the abdominal cavity, and the wound was sutured. After 5–6 weeks, the mice were sacrificed, and their spleens and livers were dissected out and embedded in paraffin. For lung metastasis model, 2×10^6^ CRC cells in 0.2 ml PBS were injected into the tail vein of male BALB/c nude mice. At the beginning to show symptoms of dying after injection, tumor in lung metastasis was removed for IVIS (Carestream Health, Inc.).

### Immunohistochemistry assay

The xenograft tumor tissue was collected and fixed in 10% formaldehyde, embedded in paraffin and then sectioned. Anti-Ki-67 antibodies (Abcam, Cambridge, UK, dilution: 1/1000) were used for immunohistochemical analyses. The secondary streptavidin-horseradish peroxidase-conjugated antibody staining was performed at room temperature, visualized in 3, 3′-diaminobenzidine (ZLI9018, ZSGBBIO, China). The slides were counterstained with hematoxylin for 15–30 s, dehydrated and fixed, sealed with the neutral gum. The TUNEL experiment was used to evaluate the apoptosis of tumors. All stained images were obtained from a light microscopy (40 × 10). Experimental results were analyzed using the software Image-Pro plus 6.0 (Media Cybernetics, Bethesda, MD, USA).

### Statistical analysis

The data were presented as the mean ± SD as indicated. Comparison between two groups was assessed using an unpaired two-tailed *t* test. A one-way analysis of variance, the chi-square test, the Fisher’s exact test was performed when appropriate. The nonparametric Mann−Whiney *U* test was employed to analyze the association of SNHG7 levels with various clinicopathologic characteristics. For statistical correlation, Spearman or Pearson correlation coefficient was used according to requirement. The overall survival was considered to be the primary endpoint. Survival curve was generated using the Kaplan−Meier method, and assessed by a log-rank test. *p* values were all two-sided and a *p* value <0.05 was considered to be statistically significant. All experiments were repeated three times and the statistical analyses were performed using GraphPad Prism (GraphPad Software, Inc., USA).

## Electronic supplementary material

Figure S1

Figure S2

Figure legends
