# Supplementary figures and images for: LncRNA SNHG7 sponges miR-216b to promote proliferation and liver metastasis of colorectal cancer through upregulating GALNT1
Source: Cell Death Dis. 2018 Jun 18;9(7):722. doi: 10.1038/s41419-018-0759-7 (PMC6006356; doi:10.1038/s41419-018-0759-7)

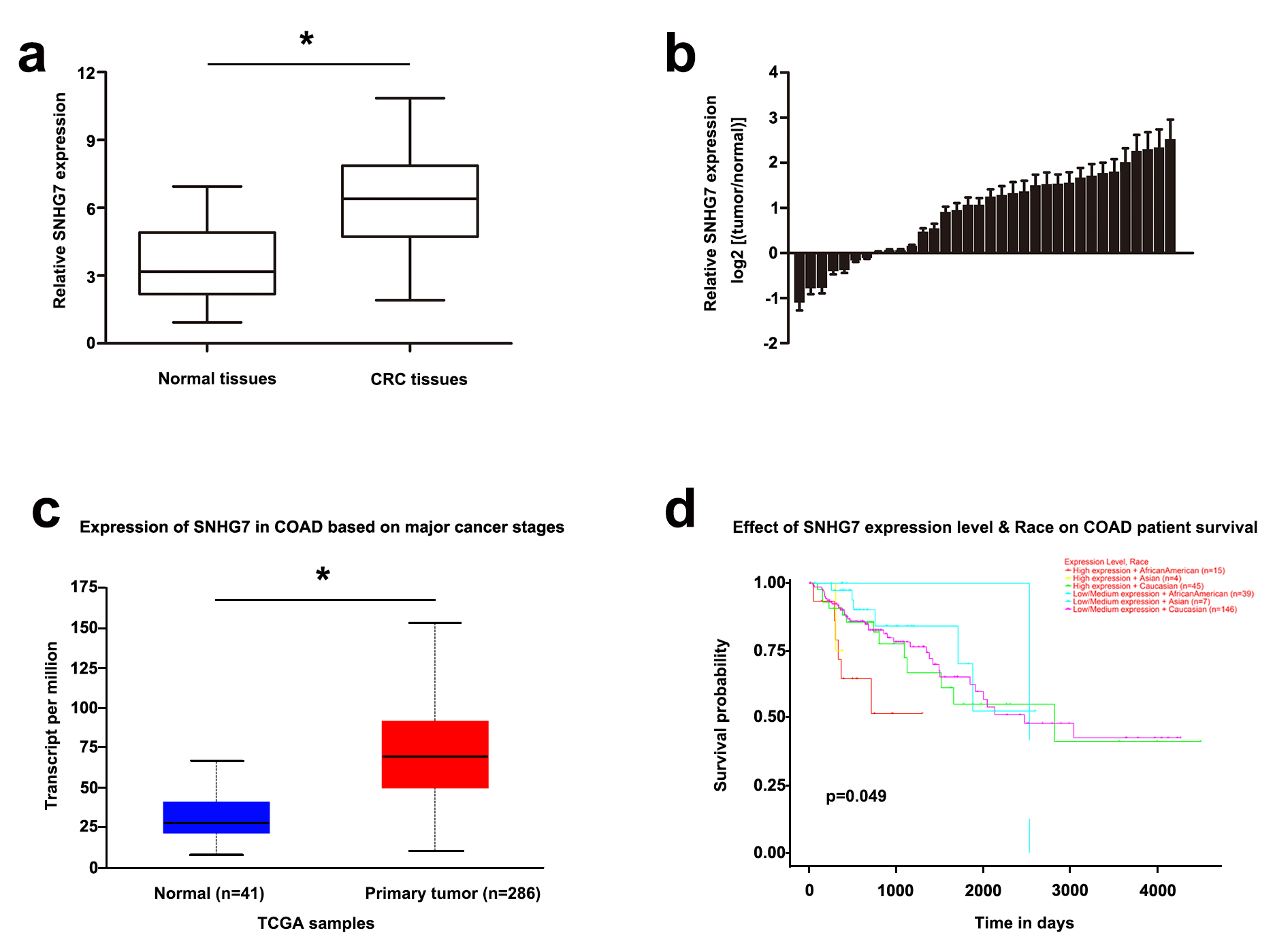

Supplement: Supplementary file 1 — Figure S1 [file 41419_2018_759_MOESM1_ESM.jpg]

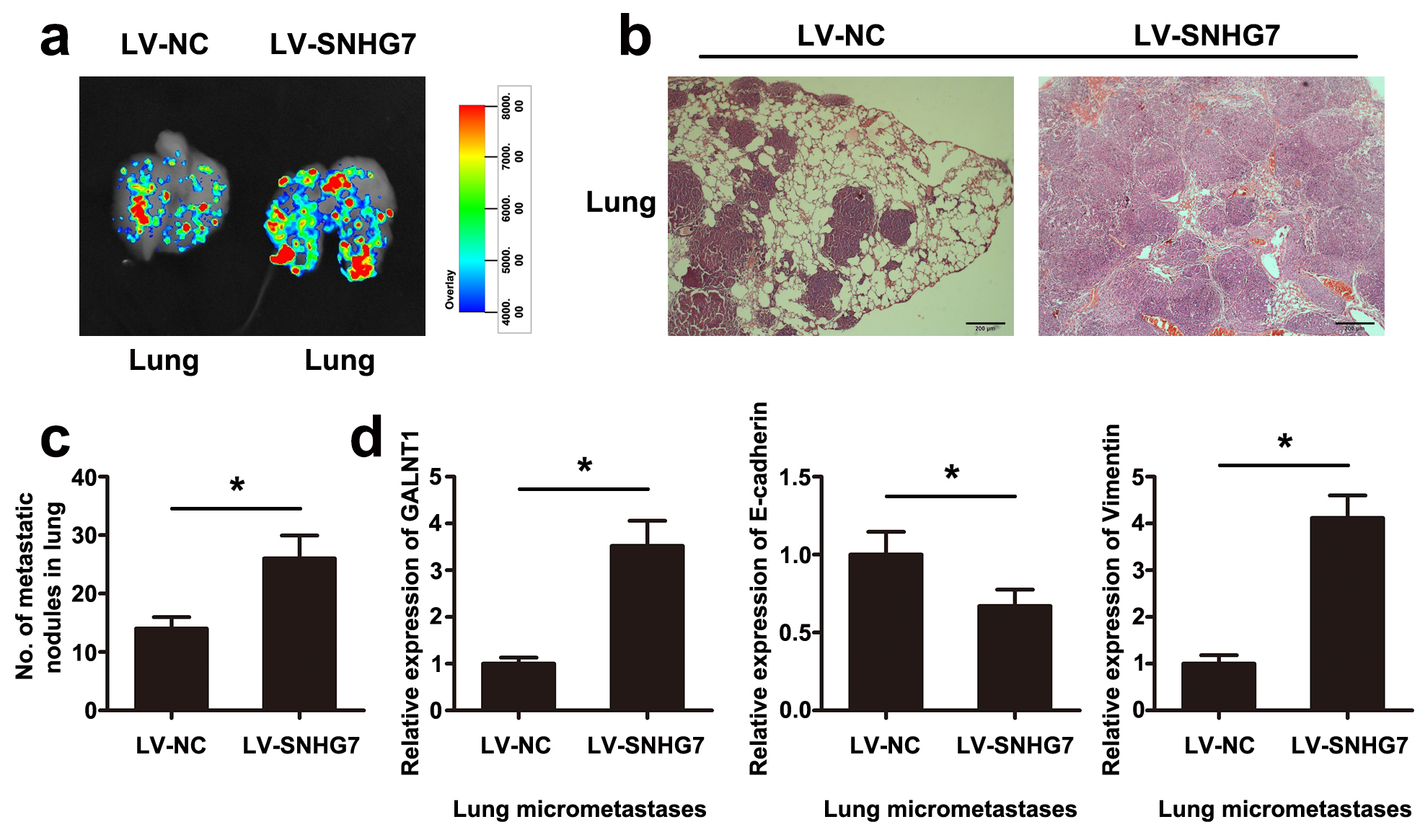

Supplement: Supplementary file 2 — Figure S2 [file 41419_2018_759_MOESM2_ESM.jpg]
