# Supplementary material for: LncRNA SNHG7 sponges miR-216b to promote proliferation and liver metastasis of colorectal cancer through upregulating GALNT1
Source: Cell Death Dis. 2018 Jun 18;9(7):722. doi: 10.1038/s41419-018-0759-7 (PMC6006356; doi:10.1038/s41419-018-0759-7)
Supplement: Supplementary file 3 — Figure legends [file 41419_2018_759_MOESM3_ESM.doc]

**Figure Legends**

**Figure S1. Expression and clinical value of SNHG7 in CRC.**

(a, b) The differential expression of SNHG7 in fresh CRC samples (n=34) and normal colon tissues (n=34) was shown. (c) Expression of SNHG7 in 286 TCGA cancer and 41 normal samples indicated that SNHG7 was obviously up-regulated in CRC. (d) The Kaplan-Meier analysis of SNHG7 expression in human colon adenocarcinoma in TCGA. The error bars in all graphs represented SD, and each experiment was repeated three times. *p < 0.05.

**Figure S2. Ectopic expression of SNHG7 promoted CRC lung metastasis in vivo.**

Representative images of GFP signals of mice in each group after tail vain injection with 2×10^6^ SW480 cells after overexpressed SNHG7. (b) H&E staining of sections from lung tumours were performed (scale bar = 200 μm) (c) The number of metastasis in the lung was determined. (d) The levels of GALNT1, E-cadherin and Vimentin mRNA in lung metastasis originating from mice were assessed by qRT-PCR. The error bars in all graphs represented SD, and each experiment was repeated three times. *p < 0.05.
